# Effects of an Individualized Active Aging Counseling Intervention on Mobility and Physical Activity: Secondary Analyses of a Randomized Controlled Trial

**DOI:** 10.1177/0898264320924258

**Published:** 2020-06-10

**Authors:** Sini Siltanen, Erja Portegijs, Katja Pynnönen, Mary Hassandra, Timo Rantalainen, Laura Karavirta, Milla J. Saajanaho, Taina Rantanen

**Affiliations:** 1 University of Jyvaskyla, Finland; 2 37786 University of Thessaly, Greece

**Keywords:** meaningful activity, physical function, life-space, autonomy, randomized controlled trial

## Abstract

**Objectives:** The aim of this study was to report preplanned secondary analyses of the effects of a 12-month individualized active aging counseling intervention on six mobility and physical activity outcomes. **Methods:** A two-arm, single-blinded randomized controlled trial was conducted among 75- and 80-year-old community-dwelling people. The intervention group (IG, *n* = 101) received counseling aimed at increasing self-selected, primarily out-of-home activity. The control group (CG, *n* = 103) received general health information. Data were analyzed with generalized estimating equations. **Results:** Physical performance improved in the IG more than that in the CG (group by time *p* = .022), self-reported physical activity increased in both groups (time *p* = .012), and autonomy in outdoor mobility declined in the IG and was enhanced in the CG (group by time *p* = .011). No change was observed for life-space mobility, proportion of persons perceiving difficulty walking 2 km, or monitored physical activity. **Discussion:** Individualized counseling aiming at increasing self-selected out-of-home activity had nonsystematic effects on mobility and positively affected physical performance only.

## Introduction

Mobility and physical activity (PA) are closely intertwined with many everyday activities of older people, such as making social visits, attending events, shopping, or running errands (Tsai et al., 2016). Increasing participation in any meaningful activity outside the home will likely increase PA and promote mobility in terms of extending life space (Barnes et al., 2007; Saajanaho et al., 2014; Saajanaho et al., 2015). In general, optimal mobility refers to the ability to move oneself safely from one place to another (Satariano et al., 2012) and allows for participation in different activities in a variety of environments. It may be viewed from such diverse perspectives as (1) the ability to perform various tasks such as walk given distances or climb stairs (Mänty et al., 2007), (2) the extent of moving about either on foot or via transportation (Peel et al., 2005), and (3) perceived ability to decide where, when, and how to move, that is autonomy in outdoor mobility (Cardol et al., 1999; Wilkie et al., 2006). These aspects can all be assessed by observing or monitoring participants or by self-reports, and combined, they will provide a more comprehensive understanding of a person’s overall mobility.

Greater life-space mobility, describing the spatial area a person moves through in daily life (Baker et al., 2003), correlates with a higher level of PA (Portegijs et al., 2015; Tsai et al., 2015), better physical performance, greater autonomy in outdoor mobility (Portegijs, Rantakokko, Mikkola et al., 2014), and fewer perceived difficulties in walking longer distances (Rantakokko et al., 2017). Viewed the other way round, mobility is a prerequisite for maintaining positive social roles, a good quality of life, and independence in old age (Patla & Shumway-Cook, 1999; Rantakokko et al., 2013; Rantanen, 2013). For example we observed that increased engagement in any out-of-home activity can improve the physical domain of quality of life, even among older people with severe mobility limitations (Rantanen et al., 2015). Furthermore, our previous studies have shown that striving for activity-related goals is associated with higher life-space mobility (Saajanaho et al., 2015) and predicts greater exercise activity (Saajanaho et al., 2014). Because people often strive to reach their goals, we expected mobility and PA to be promoted as a “side effect” of increasing the pursuit of any meaningful and self-selected activity that takes place outside the home.

This study reports preplanned secondary analyses of a randomized controlled trial of active aging counseling among community-dwelling older people. The intervention, which centered on supporting the participants’ autonomous motivation and goal setting and increasing their awareness of desirable out-of-home activities (Rantanen et al., 2019), enhanced the participants’ active aging score, although the effect was small (Rantanen et al., 2020). The aim of the present study was to test whether the individualized counseling intervention also affects physical performance, perceived difficulties in walking 2 km, life-space mobility, perceived autonomy in outdoor mobility, and self-reported and objectively monitored PA. We expected positive changes in the intervention group (IG) and no or smaller changes in the control group (CG), which received general health information by ordinary mail.

## Methods

### Design and Participants

This study reports preplanned secondary analyses of a community-based two-arm single-blinded randomized controlled trial (ISRCTN16172390), “individualized counseling for active aging—AGNES intervention.” The trial has been described in-depth in the study protocol (Rantanen et al., 2019), and the primary outcomes have been reported elsewhere (Rantanen et al., 2020). Briefly, the study participants were community-dwelling older people living in the city of Jyväskylä in Central Finland. The trial comprised two parallel groups: an IG and a CG with a 1:1 allocation ratio. Participants were recruited from among the participants of the AGNES cohort study (Rantanen et al., 2018) between October 2017 and August 2018. The inclusion criteria for the trial were willingness to participate, age 75 or 80 years, a baseline score between 52.3 and 90.0 on the University of Alabama at Birmingham Life-Space Assessment (LSA) (Baker et al., 2003), and a minimum score of 25 on the Mini-Mental State Examination (Folstein et al., 1975). In addition, participants were expected to be able to communicate. These criteria were chosen to include participants who have room for improvement in their activity levels and whose cognitive function enables compliance with the intervention. Persons participating in another ongoing intervention were excluded.

In accordance with the power calculations made for the primary outcome (Rantanen et al., 2019), 101 persons were randomly allocated to the IG and 103 to the CG. The study statistician generated the random allocation sequence with Stata 15.0 statistical software and sealed them in envelopes. Randomization was stratified by age and gender. After the pretrial data collection was completed, the study counselor opened the randomization envelopes. The flowchart of the study was previously published as part of the primary outcome article (Rantanen et al., 2019), and thus appended to this article in Appendix A. Mobility and PA were assessed pretrial (before randomization) and posttrial (at 12 months) by home interviews and activity monitoring in the free-living environment using accelerometers. Interviewers and assessors were blinded to treatment group allocation. A total of 17 persons (*n* = 10 in the IG and *n* = 7 controls) dropped out during the trial. Of these, two dropped out immediately after randomization and thus did not receive the intervention. Reasons for dropping out were unwillingness to continue (*n* = 13), health decline (*n* = 2), and death (*n* = 1). In addition, one participant’s all follow-up data were damaged.

### Intervention

The intervention’s aim was to increase self-selected meaningful activity in everyday life. Although the emphasis in the counseling and supportive material was on increasing participation in out-of-home physical and social activities, participants were supported in striving for any goals they found important (Rantanen et al., 2019). To enable personalization of the intervention, participants were profiled according to their baseline health status, social contacts, and the level of well-being, and the counseling protocol then was adjusted to their preferred activities and goals. The counseling approach was based on two major motivational theories: the self-determination theory, which highlights the importance of intrinsic and self-determined rather than external and regulated motivation behind actions (Deci & Ryan, 2000), and the theory of planned behavior, which emphasizes the role of beliefs and intentions as a basis for desirable behavior (Ajzen, 1985).

The intervention included a 90-minutes face-to-face counseling session at the research center at the beginning of the study and four shorter phone counseling sessions at months 1, 3, 6, and 9. The counseling sessions followed a semistructured protocol concerning, for example participants’ current activities, goals, and action plans. During the first session, participants were provided with supportive materials, such as an active aging information booklet, a calendar, and a newsletter (Rantanen et al., 2019). The newsletter, featuring information on the activities available in the city of Jyväskylä and stories of other participants’ success in experiencing an active life, was updated and sent to the participants every three months during the trial. The subsequent phone counseling sessions provided social support, feedback, and encouragement related to pursuing the selected goals. A trained counselor with previous experience in counseling older adults implemented the intervention.

### Control Group

Controls were mailed printed brochures and booklets related to general health at months 1, 3, 6, and 9. Brochures and booklets were obtained from different national public health associations and sorted into four themes: (1) exercise, (2) nutrition, (3) cardiovascular diseases, and (4) type II diabetes.

### Measurements

#### Physical performance

Physical performance was assessed with the Short Physical Performance Battery (SPPB), which includes tests for standing balance (feet together, semitandem, and tandem), normal gait speed over 3 m, and chair rise time (5 stands) (Guralnik et al., 1994; Rantanen et al., 2018). Each test was scored from 0 (lowest) to 4 (highest). The individual test scores were summed to form a total score (range 0–12) with higher scores indicating better physical performance.

#### Perceived walking difficulties

Perceived walking difficulties were reported for a 2-km distance with a validated question (Mänty et al., 2007). Participants were asked whether they are able to walk 2 km, and the response options were “able without difficulty,” “able with some difficulty,” “able with a great deal of difficulty,” “unable without the help of another person,” and “unable to manage even with help.” For binary logistic modeling, the responses were categorized into “no difficulties” versus “walking difficulties,” when at least some difficulties in walking were reported.

#### Life-space mobility

Life-space mobility was assessed with the University of Alabama at Birmingham Study of Aging LSA (Baker et al., 2003), which reflects the frequency and independence of mobility through different life-space levels during the preceding 4 weeks. Life-space levels start from the person’s bedroom and extend to other rooms, yard, neighborhood, town, and beyond town. Participants were asked whether they have moved in these life-space areas during the preceding 4 weeks, and if so, how often and whether they needed help from any devices or another person. A composite score (range 0–120) was used in the present analyses with higher scores indicating greater life-space mobility. The validity and reliability of the measure have been established among older people in Finland (Portegijs, Iwarsson, Rantakokko et al., 2014).

#### Autonomy in outdoor mobility

Autonomy in outdoor mobility was measured with the “autonomy outdoors” subscale of the validated Impact on Participation and Autonomy questionnaire (Cardol et al., 2001; Kersten et al., 2007). The outdoors subscale assesses the person’s self-rated possibilities to (1) visit relatives and friends, (2) make trips and travel, (3) spend leisure time, (4) meet other people, and (5) live life as he/she wants. Responses are given on a 5-point Likert scale ranging from very good (0) to very poor (4). A sum score (range 0–20) was calculated with higher scores indicating poorer autonomy. One missing item was allowed, and total scores were imputed for two persons (one at baseline and one at follow-up) based on the mean of their existing values at the same time point.

#### Self-reported PA

Self-reported PA was assessed with the second part of the Yale Physical Activity Survey, which is an interview-administered questionnaire on (1) vigorous activity, (2) walking, (3) general moving, (4) standing, and (5) sitting (Dipietro et al., 1993). For the present study, we calculated a Total Activity Summary Index (range 0–137), with higher scores indicating higher PA. The validity and reliability of the scale are moderate (Schuler et al., 2001). One participant did not have data on self-reported PA at either baseline or follow-up and thus was excluded from the analysis.

#### Monitored PA

Participants were asked to wear a triaxial accelerometer (13-bit ± 16 g, UKK RM42, UKK Terveyspalvelut Oy, Tampere, Finland) continuously for 7–10 days pretrial (Portegijs et al., 2019) and for 6 days posttrial immediately following the home interview. Only those who participated in the pretrial monitoring (*n* = 139, 68% of the total sample) were invited to participate in the posttrial monitoring. The sensor was attached on the anterior aspect of the dominant thigh (i.e. the take-off leg) with a waterproof self-adhesive film. PA, expressed as mean 24-h acceleration (milligravity, m*g*) (Rowlands, 2018), was computed as the mean high-pass filtered vector magnitude of nonoverlapping 5-s epochs (Van Hees et al., 2013). Average acceleration summarizes all movement without an intensity threshold, combines both the intensity and duration of activity into a single measure, and produces values that are directly comparable in the same wear location. Higher values indicate a greater total volume of activity. A minimum of three full days of data were required at both assessment points.

#### Background characteristics

Background characteristics included categorical variables for age, gender, perceived health, marital status, living alone, and level of education. Age and gender were drawn from the national population register and other variables self-reported. Perceived health was dichotomized as good or very good health versus moderate or poorer health. The level of education was categorized as follows: high (high school diploma or university degree), intermediate (middle school, folk high school, vocational school, or secondary school), and low (primary school or less).

### Statistical Methods

Participants’ background characteristics were examined separately in the intervention and CGs, and between-group differences were tested with the chi-square test (χ^2^). In compliance with the principles of intention-to-treat analysis (McCoy, 2017), the intervention’s effects on the different mobility and PA outcomes were tested with general estimating equation (GEE) analysis with an unstructured working correlation matrix. GEE analysis is a semiparametric method designed to work with correlated data and does not assume a normal distribution of variables (Liang & Zeger, 1986). GEE can also use information from incomplete pairs of observations and thus is suitable for use in cases of missing data in longitudinal datasets (Zhang et al., 2014). Linear models were used for continuous outcomes and binary logistic models for binary outcomes. We tested the main effects of groups and time and the interactions between these. All outcomes were analyzed in separate GEE models.

If a statistically significant group by a time effect was observed in any of the outcomes, we calculated a change score by subtracting the baseline score from the follow-up score. We used these change scores and their standard deviations to calculate effect sizes according to Cohen’s *d* formula (Cohen, 1992). Confidence intervals (CIs) for Cohen’s *d* were calculated using the formula by Lee (2016). In addition, we calculated relative improvement scores (percentual positive change) and tested between-group differences with the independent samples *t*-test and within-group differences with the paired sample *t*-test. All analyses were performed with IBM SPSS Statistics version 24.0 for Windows.

## Results

### Background Characteristics

Participants’ baseline characteristics by treatment group allocation are presented in Table 1. Of the subsample of 139 volunteer participants included in the PA monitoring at baseline, 67 were randomized into the IG and 72 into the CG. The characteristics of this subsample were found to be similar in both groups (χ^2^ = .58–.81, Table 1).

**Table 1.** Background Characteristics of the Participants by Treatment Group Allocation.

	All (*n* = 204)		PA Monitoring (*n* = 139)	
Characteristic	Intervention, *N* (%)	Control, *N* (%)	Intervention, *N* (%)	Control, *N* (%)
Age (years)				
75	75 (74)	77 (75)	49 (73)	55 (76)
80	26 (26)	26 (25)	18 (27)	17 (24)
Gender				
Female	61 (60)	63 (61)	41 (61)	46 (64)
Male	40 (40)	40 (39)	26 (39)	26 (36)
Perceived health				
Good or very good	54 (54)	64 (62)	35 (52)	41 (57)
Moderate or poorer	47 (47)	39 (38)	32 (48)	31 (43)
Marital status				
Married	61 (60)	68 (67)	46 (69)	51 (72)
Not married	40 (40)	34 (33)	21 (31)	20 (28)
Living alone				
Yes	37 (37)	33 (32)	20 (30)	20 (28)
No	64 (63)	70 (68)	47 (70)	52 (72)
Level of education				
High	28 (28)	32 (31)	18 (27)	21 (29)
Intermediate	48 (48)	56 (54)	35 (52)	39 (54)
Low	24 (24)	15 (15)	14 (21)	12 (17)

*Note*. PA = physical activity.

### Intervention Effects

**Figure 1.**
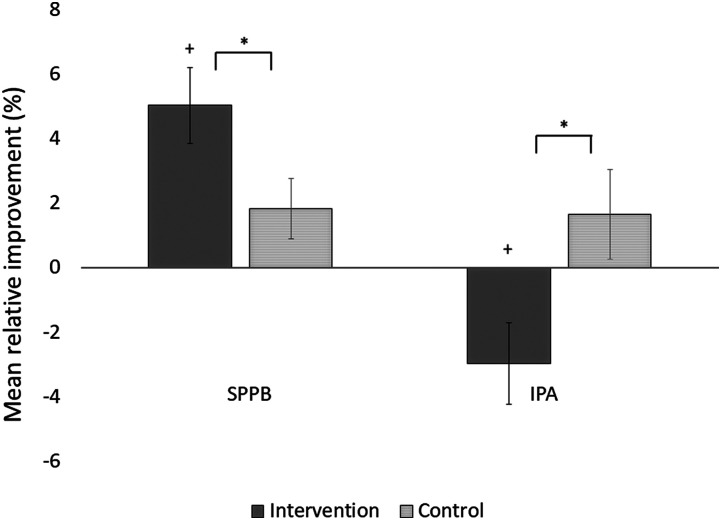
Mean relative improvements (%) with standard errors in physical performance (Short Physical Performance Battery) and perceived autonomy in outdoor mobility (Impact on Participation and Autonomy outdoors subscale) during the 12-month trial by treatment group allocation. ^∗^ Significant between-group difference, ^+^ significant within-group difference, *p* < .05.

In the GEE analyses, group, time, and group-by-time effects were not statistically significant for life-space mobility, monitored PA (average acceleration), or perceived walking difficulties (Table 2). The time effect was significant for self-reported PA (*p* = .012), indicating that PA increased both in the control and IGs. The only statistically significant group-by-time effects were observed for physical performance (*p* = .022) and perceived autonomy (*p* = .011). At baseline, the IG had poorer physical performance than the CG, but during the intervention, it reached the same level of performance as the CG and thus demonstrated greater improvement (+5.0% vs +1.8%). In addition, whereas autonomy in the CG improved during the 1-year trial, autonomy in the IG declined (+1.7% vs −3.0%, Figure 1). The effect size for physical performance was *d* = .31 (95% CI .02–.59) and for perceived autonomy *d* = .36 (95% CI .07 –.64).

**Table 2.** Means of Mobility and Physical Activity Variables by Treatment Group Allocation at Baseline and at 12-Month Follow-Up, and *p*-Values for Group, Time, and Group-By-Time Interaction Effects Tested with General Estimating Equation Analysis.

	Baseline	Follow-Up	Group	Time	Group × Time
Outcome	Mean (*SD*)	Mean (*SD*)	*p-*Value	*p-*Value	*p-*Value
Physical performance			.010	.060	.022
Intervention	10.6 (1.6)	11.2 (1.3)			
Control	11.0 (1.2)	11.2 (1.1)			
Life-space mobility			.482	.807	.409
Intervention	74.4 (9.2)	76.3 (14.5)			
Control	74.7 (9.3)	74.9 (13.6)			
Autonomy in mobility			.043	.204	.011
Intervention	4.2 (3.1)	4.8 (3.3)			
Control	4.7 (3.5)	4.3 (2.5)			
Self-reported PA^a^			.512	.012	.462
Intervention	58.9 (24.0)	65.6 (24.3)			
Control	60.3 (20.5)	65.1 (22.6)			
Average acceleration (m*g*)^b^			.401	.782	.603
Intervention	23.9 (5.9)	24.0 (7.4)			
Control	25.6 (8.4)	25.3 (9.9)			
2 km walking difficulty, no			.841	.994	.738
Intervention	81%	78%			
Control	84%	84%			

*Note*. All outcomes were analyzed in separate models. PA = physical activity, m*g* = milligravity, *SD* = standard deviation. *N* = 204 for other models.

a *n* = 203.

b *n* = 139.

## Discussion

The individualized active aging counseling intervention had no systematic effects on mobility or PA among older people. Instead, the effects were inconsistent depending on the aspect of mobility studied, as physical performance improved and sense of autonomy declined in the IG. Self-reported PA increased in both groups. No treatment effects were observed for life-space mobility, perceived difficulties in walking 2 km, or monitored PA. Although the potential association of striving for participation in meaningful activity with greater life-space mobility and PA has been established in previous observational studies (Saajanaho et al. 2015), increased active aging (Rantanen et al., 2020) did not translate to enhanced life-space mobility in the present randomized controlled trial.

Compared with controls, a small but statistically significant improvement was observed in physical performance in the IG but no change in perceived difficulty in walking 2 km. Compared with the binary perceived walking difficulty variable, the SPPB score is more sensitive to change (Ostir et al., 2002) and captures smaller improvements. Our anecdotal data from the counseling sessions suggest that some participants in the IG started resistance training or at-home exercises. These physical activities may not be fully captured by accelerometers or PA and life-space mobility questionnaires but will likely improve lower extremity performance (Rantanen, 2013).

Autonomy in outdoor mobility declined in the IG but was slightly enhanced in the CG. This was counter to our expectations because the counseling approach was autonomy-supporting and we used behavioral change techniques, such as problem solving and action planning, which aimed at helping participants obtain social and practical support in performing their selected activities. No established cut point exists for a meaningful change in the present autonomy score; however, the small decline observed may have been meaningful as it reflects participants’ own perceptions of their everyday life. The decline may potentially be explained by participants becoming aware of available activities during the intervention but not receiving practical help in engaging in them (Brandtstädter, 2009). Thus, we may have unintentionally created an imbalance in people’s aspirations for activity relative to their resources.

Although no treatment effects were found on life-space mobility or objectively monitored PA, self-reported PA increased in both groups. Compared with those participating in the self-reports only, those also involved in the PA monitoring reported fewer depressive symptoms and a higher level of PA (Rantanen et al., 2019), thus potentially possessing less room for improvement in their activity levels. However, self-reported PA increased similarly among both subgroups, making selection bias an unlikely explanation for this finding. Instead, it has been found that self-reports of PA are vulnerable to bias related to social desirability and approval (Adams et al., 2005) and may lead to overestimations of activity (Steene-Johannessen et al., 2016). The similar change in the intervention and CGs may be due to the Hawthorne effect, that is the fact that people often act differently simply because they are being studied rather than because of the intervention they are receiving (Becker et al., 2003).

Overall, these findings imply that changing everyday behaviors is challenging. According to anecdotal data from the counseling sessions, many of our participants preferred striving to maintain their current situation and activity rather than setting new activity goals. This maintenance rather than growth orientation in goal pursuit is common in old age and correlates with well-being (Ebner et al., 2006) but may have led to invariability in the present outcomes. In addition, although the counseling was centered on promoting out-of-home activity, some people rather set goals related to more sedentary and at-home activities. Furthermore, as we detected only small increases in the trial’s primary outcome variable of overall activity (Rantanen et al., 2020), it is understandable that the changes in mobility and PA outcomes were also modest. Finally, recruitment for the present trial was based on a population-based probability sample, which should reduce selection bias. However, activity studies of this kind tend to attract healthy, active, and interested people (Portegijs et al., 2019), which, in turn, may lead to participants with higher than expected levels of functioning and activity.

### Strengths of the Study

The strengths of this study include the randomized controlled trial design and the use of validated and established measures. Unlike in many earlier studies, we considered various mobility and PA outcomes, including both subjective and objective measures. In addition, our population-based sample contained both men and women at ages that are vulnerable to functional decline. Finally, we had barely any missing data and low attrition, as only a few participants dropped out of the rather long trial. Furthermore, we applied GEE analysis, which also takes unpaired observations into account (Zhang et al., 2014) in examining the causal associations.

### Study Limitations

A notable limitation in this study was the use of only two assessment points: baseline and 12 months thereafter. Thus, we do not know how mobility or PA might have changed in between these time points. For example it is possible that the intervention positively affected mobility and/or PA immediately after the face-to-face counseling, but as face-to-face contact was replaced by phone calls and became less frequent, such effects diminished. In addition, we may have failed to recruit enough people with early phase decline in health and activity, as such individuals would likely have had more room and motivation for increasing their level of activity. Finally, it should be noted that PA patterns and life-space mobility may be highly variable due to the normal variation in everyday life (Terwee et al., 2010) or other factors such as weather conditions (Portegijs, Iwarsson, Rantakokko et al., 2014) that make it harder to detect change in longitudinal data with momentary assessments of mobility.

## Conclusion

We found that individualized counseling centered on increasing self-selected meaningful activity outside the home has inconsistent effects on mobility and PA. Although modest positive changes were seen in physical performance in the IG, perceived autonomy in outdoor mobility declined and no divergent changes were observed in the other outcome variables. This suggests that promoting mobility and PA is no easy task in population-based studies targeting increased participation in a variety of activities. Because earlier studies on individualized PA counseling have reported more positive results for physical function (Mänty et al., 2009) and PA (Rasinaho et al., 2012), we may deduce that future interventions tailored specifically for increasing participation in mobility-related and physical activities could yield greater positive effects than those reported here.
